# Identifying subtypes of bipolar disorder based on clinical and neurobiological characteristics

**DOI:** 10.1038/s41598-021-96645-5

**Published:** 2021-08-24

**Authors:** Yen-Ling Chen, Pei-Chi Tu, Tzu-Hsuan Huang, Ya-Mei Bai, Tung-Ping Su, Mu-Hong Chen, Yu-Te Wu

**Affiliations:** 1 Institute of Biophotonics, National Yang Ming Chiao Tung University, No. 155, Sec. 2, Linong St., Taipei, 112 Taiwan; 2 Brain Research Center, National Yang Ming Chiao Tung University, Taipei, 112 Taiwan; 3 Department of Medical Research and Education, Taipei Veterans General Hospital, Taipei, 112 Taiwan; 4 Department of Psychiatry, Taipei Veterans General Hospital, Taipei, 112 Taiwan; 5 Division of Psychiatry, Faculty of Medicine, National Yang Ming Chiao Tung University, Taipei, 112 Taiwan; 6 Institute of Philosophy of Mind and Cognition, National Yang Ming Chiao Tung University, Taipei, 112 Taiwan; 7 Department of Psychiatry, Cheng-Hsin General Hospital, Taipei, 112 Taiwan

**Keywords:** Bipolar disorder, Biomarkers, Epidemiology, Translational research

## Abstract

The ability to classify patients with bipolar disorder (BD) is restricted by their heterogeneity, which limits the understanding of their neuropathology. Therefore, we aimed to investigate clinically discernible and neurobiologically distinguishable BD subtypes. T1-weighted and resting-state functional magnetic resonance images of 112 patients with BD were obtained, and patients were segregated according to diagnostic subtype (i.e., types I and II) and clinical patterns, including the number of episodes and hospitalizations and history of suicide and psychosis. For each clinical pattern, fewer and more occurrences subgroups and types I and II were classified through nested cross-validation for robust performance, with minimum redundancy and maximum relevance, in feature selection. To assess the proportion of variance in cognitive performance explained by the neurobiological markers, multiple linear regression between verbal memory and the selected features was conducted. Satisfactory performance (mean accuracy, 73.60%) in classifying patients with a high or low number of episodes was attained through functional connectivity, mostly from default-mode and motor networks. Moreover, these neurobiological markers explained 62% of the variance in verbal memory. The number of episodes is a potentially critical aspect of the neuropathology of BD. Neurobiological markers can help identify BD neuroprogression.

## Introduction

Bipolar disorder (BD) is a mood disorder with episodic fluctuations in affection and a leading cause of disability worldwide, affecting > 1% of the global population^1^. BD is reportedly characterized by neurobiological deficits^2^, as revealed through group-level inferences. Rather than group level, classification is a type of supervised machine learning approach used to predict classes of data and analyze the neuropathology of diseases at an individual level. However, it is difficult to adequately classify patients with BD, owing to the heterogeneity in common psychiatric disorders^3,4^.

Identification of individuals with subtypes reduces this heterogeneity and potentially increases the predictive accuracy of psychiatric disorders^5–7^. Dwyer et al.^6^ reported that classification accuracies for subgroups were higher than that for the entire group. Yang et al.^8^ reported that the treatment of heterogeneous patients as a unitary group may reduce the classification rate during binary classification analysis. Approaches to identify patient subtypes include the assessment of overall brain morphology^6,9^, brain region activation^10^, functional connectivity^11^, and white-matter integrity^12^. These neuroimaging approaches have been suggested as the intermediate phenotype between two psychiatric disorders or patient–control pairs^13–15^. Furthermore, these subtypes differ in their responses to treatment^11,16^. Nevertheless, subtypes derived from neurobiological markers reported by studies with a transdiagnostic psychiatric approach were not similar to previously reported diagnostic groups^17^. Moreover, patient subtypes determined using previously reported unsupervised learning approaches were barely distinguishable by clinical patterns, including symptoms and treatment responses, and clinical patterns were merely compared at the group level or were defined by featured clinical patterns^18,19^.

Consequently, categorization of patients into predefined subtypes based on clinical features served as a potential approach to improve classification performance and better understand the neuropathology of BD^20^, especially the precise neurobiological basis of a BD subtype. Based on the mood episodes a patient experiences, BD is clinically categorized into two common subtypes: bipolar I disorder (BDI) and bipolar II disorder (BDII). However, clinical dimensions other than diagnosis (i.e., BDI and BDII) were considered as approaches for better understanding the pathophysiology of mood disorders^5,21^. For example, among the clinically defined subtypes, a review article indicated that corpus callosum function may be a promising biomarker for patients with BD and a history of psychosis; however, the differentiation of BDI and BDII has yielded inconsistent outcomes^22^. Furthermore, the extent to which patients with BD can be classified on the basis of neuroimaging abnormalities remains unclear, likely due to the heterogeneity among such patients and some redundant predictors in their abnormalities.

Because studies on both precise phenotypic delineation of BD and the neurobiological representation of subtypes are underway, this study aimed to investigate the differentiated subtypes through multimodal neuroimaging, including the analysis of overall brain morphology and functional connectivity, and the essential markers for BD subtypes categorized by clinical dimensions, including the number of episodes and hospitalizations and the history of suicide and psychosis. Furthermore, the diagnostic subtypes, namely BDI and BDII, were also comparatively analyzed.

## Results

### Demographic characteristics of the BD subgroups based on clinical patterns and diagnostic subtypes

To numerically balance individual pairs of fewer and more occurrences groups with different clinical patterns, patients were categorized on the basis of low (n = 43, less than 10 times) and high (n = 55) numbers of episodes, never (n = 34) or more than one (n = 35) instance of hospitalization, having (n = 45) or not having (n = 53) attempted suicide, and having (n = 43) or not having (n = 54) a history of psychosis. Table 1 and Supplementary Table S1 summarize the results of the comparison of demographic data among pairs of fewer and more occurrences groups, along with diagnostic subtypes (BDI/BDII: n = 51/43).

**Table 1:** Demographic data of groups with fewer and more occurrences of clinical patterns and diagnostic subtypes.

	Fewer occurrences group	More occurrences group	*p*-value
**The number of episodes**			
Sample size	43	55	
Age	34.05 ± 14.386	41.45 ± 12.176	0.0069*
Sex			0.0226*
Male (%)	8 (18.6)	22 (40.0)	
Female (%)	35 (81.4)	33 (60.0)	
YMRS	2.80 ± 4.267	3.60 ± 5.138	0.4236
MADRS	10.17 ± 9.864	12.70 ± 10.689	0.2429
PANSS	41.95 ± 13.090	41.08 ± 12.911	0.7465
**The number of hospitalizations**			
Sample size	34	35	
Age	33.71 ± 13.962	43.71 ± 11.372	0.0017*
Sex			0.2333
Male (%)	9 (26.5)	14 (40.0)	
Female (%)	25 (73.5)	21 (60.0)	
YMRS	4.62 ± 5.726	2.64 ± 4.879	0.1328
MADRS	15.38 ± 10.583	9.00 ± 10.476	0.0157*
PANSS	44.65 ± 13.470	39.09 ± 13.185	0.0928
**Whether attempting suicide**			
Sample size	53	45	
Age	37.09 ± 14.688	39.51 ± 12.304	0.3845
Sex			0.0968
Male (%)	20 (37.7)	10 (22.2)	
Female (%)	33 (62.3)	35 (77.8)	
YMRS	2.92 ± 4.797	3.67 ± 4.761	0.4554
MADRS	9.654 ± 10.031	14.00 ± 10.371	0.0425*
PANSS	40.62 ± 13.010	42.50 ± 12.902	0.4852
**Whether having the history of psychosis**			
Sample size	43	54	
Age	37.42 ± 14.492	38.98 ± 13.085	0.5787
Sex			0.7566
Male (%)	14 (32.6)	16 (29.6)	
Female (%)	29 (67.4)	38 (70.4)	
YMRS	3.07 ± 5.330	3.33 ± 4.325	0.7942
MADRS	10.62 ± 10.138	12.39 ± 10.672	0.4169
PANSS	40.74 ± 12.601	42.10 ± 13.402	0.6181

	BDI	BDII	*p*-value
Sample size	51	43	
Age	40.98 ± 13.804	35.16 ± 13.026	0.0395*
Sex			0.0982
Male (%)	20 (39.2)	10 (23.3)	
Female (%)	31 (60.8)	33 (76.7)	
YMRS	3.41 ± 5.435	3.20 ± 4.137	0.8373
MADRS	9.69 ± 9.732	13.54 ± 10.736	0.0786
PANSS	40.84 ± 12.795	42.56 ± 13.710	0.5393

*p < 0.05.

*YMRS* Young Mania Rating Scale, *MADRS* Montgomery–Åsberg Depression Rating Scale, *PANSS* Positive and Negative Syndrome Scale, *UKU* UKU Side Effects Rating Scale, *PSP* Personal and Social Performance Scale, *GAF* Global Assessment of Functioning, *BDI* bipolar type 1 disorder, *BDII* bipolar type 2 disorder.

### Performance of classification of the BD subgroups based on clinical patterns and diagnostic subtypes from among multimodal neurobiological markers

**Figure 1:**
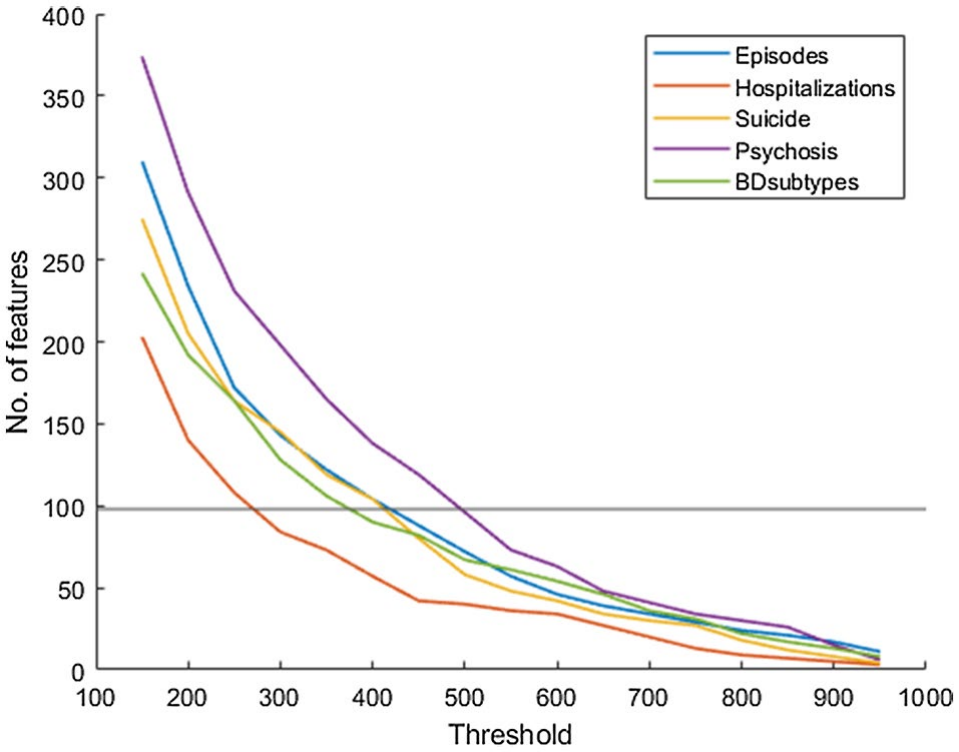
Number of features with different thresholds.

Through the bootstrapping approach, 1000 sets of features selected through minimum redundancy maximum relevance (mRMR^23^) were obtained. The number of features accumulated from more than one threshold range (150–950-folds with 50-fold increments) for classifying four different types of clinical patterns and the diagnostic subtypes are displayed in Fig. 1. To prevent the “curse of dimensionality” in accordance with the sample size (i.e., 98 patients) and the total number of features selected through mRMR, the number of features accumulated from > 400–800-folds was used for training SVM classification models. However, the features accumulated from > 500–800-folds were utilized for training SVM classification models on the basis of a history of psychosis (see Fig. 1).

**Figure 2:**
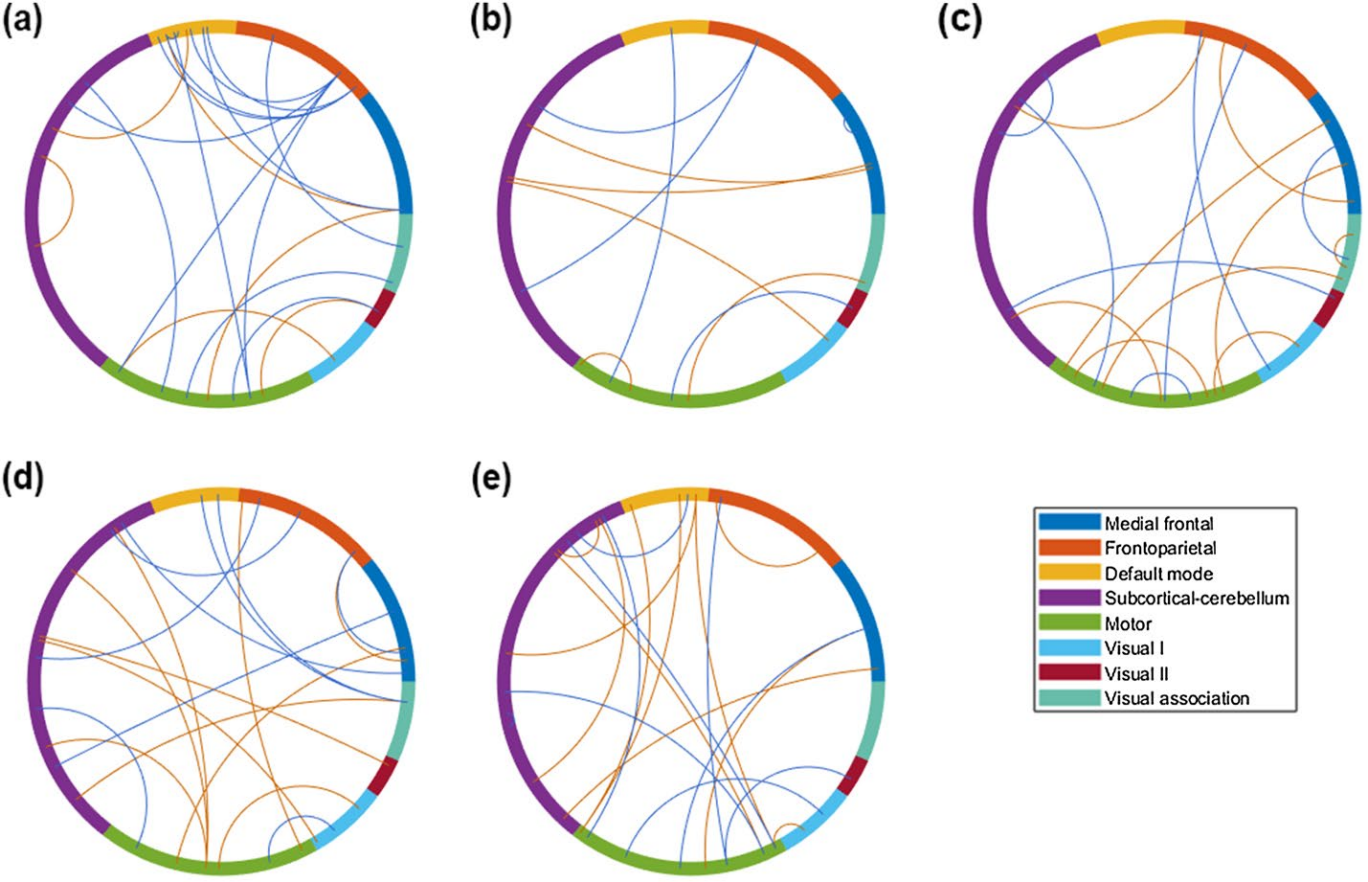
The major features of the classification analyses for the clinical patterns. After 100 processes of minimum redundancy maximum relevance selection with the features selected through with the threshold of the most optimal mean performance, the major features were selected over 90 times during the outer loop of the nested cross-validation. The illustration uses eight networks of Shen’s 268-region parcellation. The red lines represent the connectivity of the more occurrences group being higher than that of the fewer occurrences group, and the blue lines represent the opposite direction. (**a**) The major features of the classification for the number of episodes. (**b**) The major features of the classification based on the number of hospitalizations. (**c**) The major features of the classification based on suicide attempts. (**d**) The major features of the classification based on the history of psychosis. (**e**) The major features of the classification based on diagnostic subtypes. The red lines represent the connectivity of BDI being higher than that of BDII, and the blue lines represent the opposite direction.

**Figure 3:**
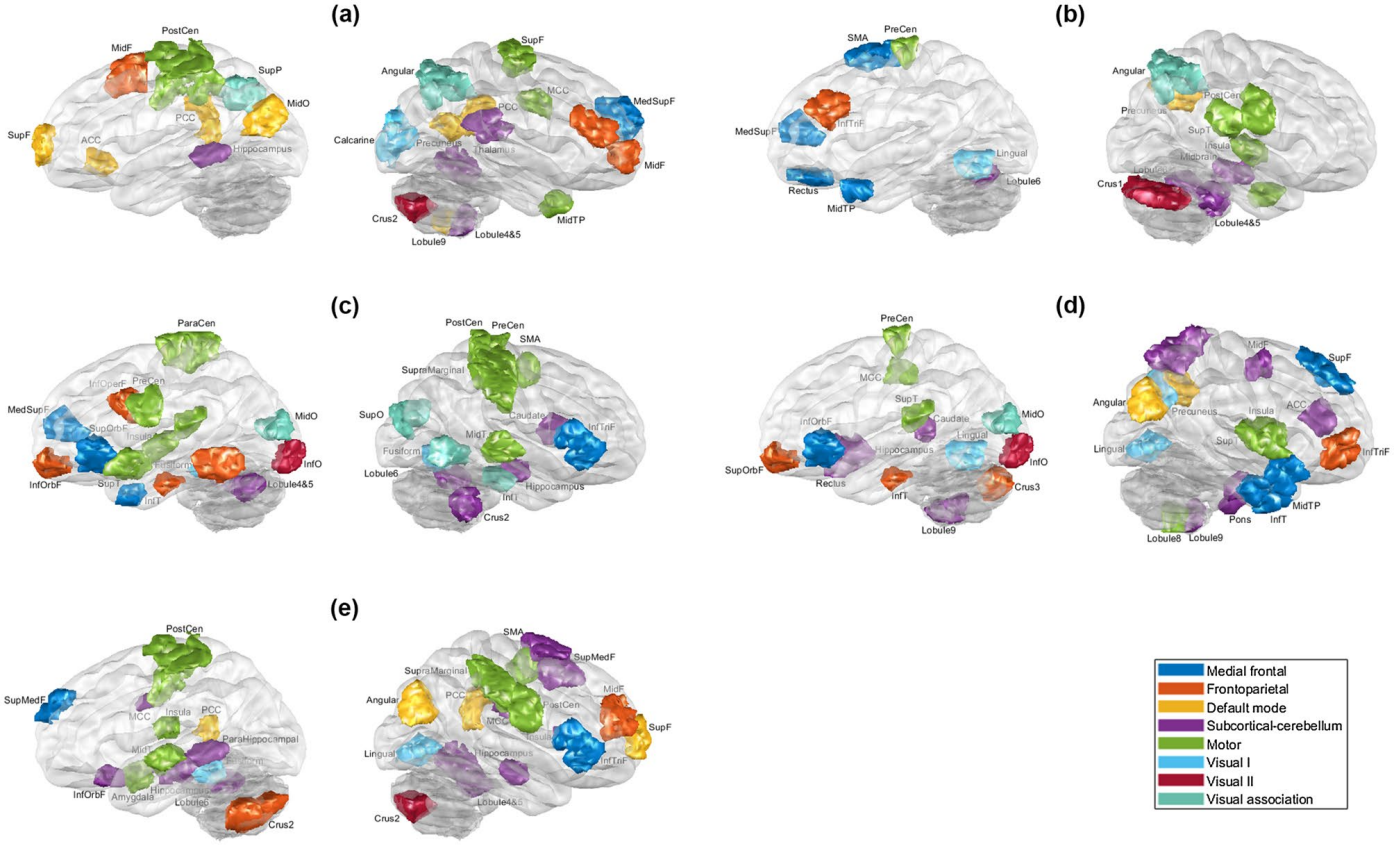
The involved regions of major features of the classification analyses for fewer and more occurrences groups based on the number of episodes, the number of hospitalizations, suicide attempts, and having psychosis or not, which were selected more than 90 times after 100 times of processing of minimum redundancy maximum relevance selection during the outer loop of the nested cross-validation, as illustrated on the glass brain. (**a**) The involved regions of major features of the classification for the number of episodes. (**b**) The involved regions of major features of the classification based on the number of hospitalizations. (**c**) The involved regions of major features of the classification based on suicide attempts. (**d**) The involved regions of major features of the classification based on the history of psychosis. (**e**) The involved regions of major features of the classification based on diagnostic subtypes. (*ACC* anterior cingular cortex, *InfO* inferior occipital cortex, *InfOperF* inferior opercular frontal cortex, *InfOrbF* inferior orbitofrontal cortex, *InfT* inferior temporal cortex, *InfTriF* inferior triangular frontal cortex, *MCC* middle cingular cortex, *MidF* middle frontal cortex, *MidO* middle occipital cortex, *MidTP* middle temporal pole, *ParaCen* paracentral lobule, *PCC* posterior cingular cortex, *PostCen* postcentral cortex, *PreCen* precentral cortex, *SMA* supplementary motor area, *SupF* superior frontal cortex, *SupMedF* superior medial frontal cortex, *SupO* superior occipital cortex, *SupOrbF* superior orbitofrontal cortex, *SupP* superior parietal cortex, *SupT* superior temporal cortex).

After 100 nested cross-validations conducted in accordance with the threshold range, the best mean performance, which was the highest mean testing accuracies of 100 performances with a specific threshold, was determined for each clinical pattern or diagnostic subtype. For classification based on the number of episodes, the most optimal mean performance was observed at a threshold of 750-folds with 34 features in total; number of hospitalizations, 650-fold threshold with 27 features in total; suicide attempts, 500-fold threshold with 58 features in total; history of psychosis, 550-fold threshold with 63 features in total; and diagnostic subtypes, 550-fold threshold with 61 features in total. All the features were not overlapped between each paired subgroup. Moreover, features with the most optimal mean performance for diagnostic subtypes and these clinical patterns, except the subgroup categorized on the basis of a history of suicide, only displayed functional connectivity but no brain morphology after feature selection. The most optimal mean performance on classification analyses of four different clinical patterns and of diagnostic subtypes are presented in Table 2. The most optimal performance determined from four different clinical patterns and diagnostic subtypes was > 70%. Both the accuracies of classifying groups based on the number of episodes and diagnostic subtypes were significantly different with other paired subgroups. Furthermore, Fig. 2 (only the features extracted from functional connectivity) and Supplementary Table S2 indicate the major features, including those selected from the threshold for the best mean performance also selected over 90 times on mRMR within 100 nested cross-validations, for classifying fewer and more occurrences groups of four different clinical patterns and for BDI and BDII. Most major features for differentiating fewer and more occurrences groups in accordance with the number of episodes were evident in the default mode network (DMN) and the motor network (MON); the number of hospitalizations, the MON, and subcortical and cerebellar regions (SC); suicide attempts, the medial frontal network (MFN) and MON; and those for differentiating BDI and BDII, the DMN, MON, and SC. Specifically, major features for differentiating subtypes according to the number of episodes were primarily in functional connectivity between the DMN and FPN and between the MON and almost all the other networks; the number of hospitalizations, between the SC and MFN and between the SC and FPN; suicide attempts, between the MON and MFN and within the MON; and those for differentiating BDI and BDII, between the MON and SC. Moreover, the major features for differentiating the two groups based on history of psychosis were distributed to the frontoparietal network (FPN), MFN, MON, and SC. Furthermore, Fig. 3 illustrates major features of the four clinical patterns and clinical subtypes on the glass brain in involved regions and the networks they appertain to.

**Table 2:** Model performance for classifying fewer and more occurrences groups divided from clinical patterns.

Clinical patterns	Performance			
	Accuracy	Sensitivity ^a^	Specificity ^b^	AUC
Low (n = 43) vs. High (n = 55) number of episodes	73.60 ± 3.78	75.43 ± 3.45	72.77 ± 5.71	0.795 ± 0.0381
Never (n = 34) vs. more than one time (n = 35) of hospitalizations	70.75 ± 6.07	72.11 ± 6.48	74.31 ± 6.71	0.744 ± 0.0716
Had (n = 45) vs. Hadn’t (n = 53) attempted suicide	71.81 ± 4.09	73.35 ± 5.62	72.75 ± 4.24	0.774 ± 0.0383
Had (n = 54) vs. Hadn’t (n = 43) the history of psychosis	71.08 ± 3.86	68.12 ± 4.96	74.76 ± 4.28	0.774 ± 0.0347
BDI (n = 51) vs. BDII (n = 43)	75.42 ± 4.22	76.37 ± 6.41	76.36 ± 3.84	0.824 ± 0.0428

*AUC* area under the ROC curve, *BDI* bipolar type 1 disorder, *BDII* bipolar type 2 disorder.

^a^Sensitivity of more occurrences group or BDI.

^b^Specificity of more occurrences group or BDI.

### Multiple linear regression between verbal memory and features for classification of groups based on clinical patterns and diagnostic subtypes

The predictive ability of the selected features of paired subgroups on each task of Word List Test (WLT), including immediate and delayed free recall, learning slope, recognition, and retention of lists of words, was examined. After correcting the false discovery rate for multiple comparisons, the associations were significant between retention and the selected features on the basis of the number of episodes (*R*^2^ = 0.62, *q* = 0.0452), between immediate free recall and those based on the number of hospitalizations (*R*^2^ = 0.68, *q* = 0.0484), and between recognition and those based on the number of hospitalizations (*R*^2^ = 0.73, *q* = 0.0185).

### Potential influence of various clinical confounding factors on patient classification based on clinical patterns and diagnostic subtypes

After correcting the false discovery rate for multiple comparisons, no significant association among the selected features for differentiating paired subgroups with either illness duration or mood states was observed. Furthermore, only the features selected from bootstrapping approach but not major features were correlated with symptom scores (i.e., YMRS, MADRS, and PANSS). Regarding the number of hospitalizations, significant correlations were observed between MADRS score and the functional connectivity of the right insula and right middle frontal cortex; a history of suicide, between YMRS and the functional connectivity of the right inferior temporal cortex and right supramarginal cortex; and BDI/BDII, between MADRS score and the functional connectivity of the right middle cingulate cortex and right angular gyrus. Furthermore, no significant differences were observed among most selected features for differentiating pairs of subgroups between the patients receiving treatment with atypical antipsychotics, antidepressants, and/or mood stabilizers or those not receiving treatment. However, for the features for classification based on a history of psychosis, a significant difference between patients taking antidepressants in the functional connectivity of the left and right lobule VIII of cerebellum was observed; and BDI/BDII, between the patients taking atypical antipsychotics in the functional connectivity of the left middle frontal cortex and right superior medial frontal cortex. These results suggest that few clinical confounding factors affected the classification and regression of patients based on clinical patterns and diagnostic subtypes.

In addition, classification was also conducted for differentiating BD above (n = 50) or under (n = 48) the mean age, since age was not matched between most pairs of subgroups. As a result, the number of features accumulated from 10 to 50 with 20-fold increments because a smaller number of them were selected from bootstrapping approach. The most optimal mean performance was observed at a threshold of 50-folds with 15 features in total, and the accuracy, sensitivity, specificity, and area under the receiver operating characteristic curve (AUC) were 52.66 ± 3.26, 53.36 ± 3.69, 52.56 ± 9.10, and 53.02 ± 4.60, respectively, showing that it is unable to use imaging data controlled by age and sex for classifying BD with above or under the mean age.

## Discussion

In this study, BD patient subgroups were better categorized on the basis of diagnostic subtypes and the number of episodes by using neurobiological markers. The mean accuracy of classification between the patients with type I or type II was 75.42%, and that between the patients with a high or low number of episodes was 73.60%. However, only the features for classifying the subgroups based on the number of episodes were independent of clinical confounding factors, including duration, symptom scores, mood states, and medication. Moreover, these neurobiological markers for differentiating high or low number of episodes can explain 62% of the variance in verbal memory. Therefore, the subgroups based on the number of episodes rather than diagnosis have the potential to be clinically discernible, neurobiologically distinguishable, and functionally predictable BD subtype.

### Classification of subgroups based on epidemiological and clinical factors

Classifying patients with BD on the basis of epidemiological (such as the number of hospitalizations) and clinical subgroups rather than from the entire BD patient group plays an important role in understanding the core clinical dimensions of BD^20^. The selection of pertinent clinical dimensions correlated with neurobiological factors was considered critical owing to potentially significant differences resulting from clinical heterogeneity. Furthermore, the application of machine learning approaches to study psychiatric disorders is challenging for both clinical differentiation or diagnosis and is unsuitable for the identification of neurobiological markers^24^. The clinical challenges result from the higher prevalence of depressive symptoms rather than mania or hypomania^25^ due to subthreshold BD symptoms^26^. Moreover, these challenges have rendered the pathophysiological factors associated with the current differentiation controversial^27^. However, the use of clinical patterns may be a more suitable approach for categorizing patients with BD rather than for diagnostic differentiation. As revealed in this study, both the performance of distinguishing patients with BDI and BDII and that for the classification of subgroups based on the number of episodes were better than the classification of other paired subgroups. However, the proportion of variance in verbal memory explained by the neurobiological markers for the number of episodes was high. Moreover, even though the classification of patients on the basis of the number of hospitalizations was lower than the above two paired subgroups, the proportion of variance in verbal memory explained by these neurobiological markers was also high. Hence, the groups based on epidemiological and clinical features may serve alternative clinical dimensions and subcategories. Additionally, clustering BD based on the number of episodes in order to improve within-group homogeneity was consistent with a review study^28^, which indicated that BD with a phasic-recurrent course might be a clinical-biological subgroup of BD, along with the evidence of alteration in immune-inflammation and in the white matter of limbic regions.

### Classification of the patients with a high or low number of episodes reflects BD as a neuroprogressive disorder

The satisfactory performance of classification herein indicates the importance of differentiating patients on the basis of the number of episodes when assessing recurrence effects at an individual level. Because BD is characterized by mania, hypomania, and depressive episodes, BD progression involves periods of not only remission but also recurrence, particularly owing to poor treatment responses^29^. Along with its disease course, BD is characterized by an increase in the frequency and severity of episodes^30^ and reduction in neuroprotective factors, resulting in a more prominent negative impact of episodes^31^. Furthermore, patients with multiple episodes potentially encounter progressive neuropathological changes when mood episodes relapse^32,33^. Previous studies have reported that neuropathological progression includes deactivation of fronto–limbic–striatal regions^34^.

The results of classification based on low or high number of episodes of the present study could achieve satisfactory performance because the selected features, which were primarily in the DMN and MON, were specific to regions associated with the neuropathology of BD. As reported previously, patients with BD present dysconnectivity in the DMN^35–37^, which is a task-negative network with greater activity at rest than during goal-directed tasks^38^. Moreover, consistent with Gong et al.^36^, dysconnectivity of two key regions in the DMN, the precuneus and posterior cingulate cortex, was observed; these disconnections are considered the core pathophysiological features for differentiating BD from schizophrenia^39^, from among the selected major features of the classification. Furthermore, two studies of the Research Domain Criteria have indicated that the within-network connectivity of the MON and the between-network connectivity of the MON and other subcortical regions, such as the caudate, thalamus, and cerebellum, play an essential role in general psychopathology, cognitive dysfunction, and impulsivity across multiple psychiatric disorders^5,40^. Moreover, Conio et al.^41^ demonstrated that dopaminergic and serotonergic pathways modulate the balance of functional connectivity in sensorimotor regions and the DMN. Previous studies also indicated that changed dopamine and serotonin transmission, which modulated activity of sensorimotor regions and the DMN and balance between these networks through the connection from basal ganglia and thalamus, resulted in excitation or inhibition of affectivity, psychomotricity, and thought^42,43^. Altered biochemical modulation induces dysconnectivity in these networks, which results in the occurrence of different states of BD. Consequently, DMN and MON dysconnectivity may not only represent the neuropathological features of the entire BD group but also the neuroprogressive features for patients with multiple episodes.

### Factors potentially contributing to the classification of fewer and more occurrences groups of other clinical patterns

The inferior mean performance in distinguishing patients on the basis of the number of hospitalizations, suicide attempts, and the history of psychosis may result from the premise that the neurobiological markers are not the major and robust features for classification. For example, Li et al.^44^ reported that rehospitalization was associated with a maximal score of YMRS item 8, the number of previous hospitalizations, nonremittance at discharge, and discharge against medical advice. Furthermore, previous studies have reported that suicide attempts of patients with BD are associated with factors other than neurobiological markers, including trait aggression or impulsivity, early onset, frequent depressive episodes, having a history of rapid cycling, and a family history of suicide^45–48^, which may be supported by the psychological, occupational, and social functioning impairments associated with suicide attempts. Furthermore, Burton et al.^49^ and Keck et al.^50^ suggested that a history of psychosis may not represent a more severe subtype among patients with BD, having studied a relatively large-size cohort. Therefore, other factors may influence the classification of fewer and more occurrences groups based on these clinical patterns.

### Limitations

Several limitations should be considered with respect to this study. First, the effects of medication were not thoroughly reversed insofar as most patients recruited herein received pharmacotherapy including atypical antipsychotics, antidepressants, and mood stabilizers. Nevertheless, the most features for the classification may not be influenced by either various types of medication in this study. Second, during feature selection for differentiating the fewer and more occurrences groups based on clinical patterns, the step was not conducted only for the training set. Hence, the results did not prevent the issue of double dipping^51^. However, feature selection only for the training set during nested cross-validation can effectively reduce the difference between training and testing accuracies in the study. Third, age and sex were not matched between some pairs of subgroups because the cohort sizes were balanced between pairs of subgroups. To resolve this issue, we controlled the age and sex covariates before feature selection and model training. In addition, the complementary analysis also demonstrated that the age- and sex-controlled imaging data misclassified the paired subgroup clustered by age.

## Material and methods

### Participants

In total, 112 patients with BD, including inpatients and outpatients, were recruited from Taipei Veterans General Hospital, Taipei, Taiwan. An experienced physician verified the patients’ diagnoses through structured clinical interviews according to the *Diagnostic and Statistical Manual of Mental Disorders, Fourth Edition*^52^. The exclusion criteria included a diagnosis of a neurological disorder or any other disorders impacting cerebral metabolism, substance abuse or chemical dependence history during the past 6 months, and a history of head injury with a sustained loss of consciousness and/or neurological sequelae. Current symptoms of patients with BD were assessed using the Young Mania Rating Scale (YMRS), the Montgomery–Åsberg Depression Rating Scale (MADRS), and the Positive and Negative Syndrome Scale (PANSS). Demographic characteristics of patients were collected by interviews and medical record review. Regarding medication, the patients had been taking various atypical antipsychotics, antidepressants, and mood stabilizers before participating in this study. All participants provided written informed consent to confirm their participation in this study after the procedures were completely explained to them. This study was approved by the Research Ethics Committee of Taipei Veterans General Hospital and the Institutional Review Board of National Yang-Ming University, and was conducted in accordance with the tenets of the Declaration of Helsinki.

### Resting-state functional and structural magnetic resonance imaging

Scanning was performed using a 3.0-T GE magnetic resonance imaging (MRI) scanner (GE Healthcare Life Sciences, Little Chalfont, UK) with a quadrature head coil at the Taipei Veterans General Hospital. Anatomical whole-brain image volumes were determined using a sagittal magnetization-prepared rapid acquisition gradient-echo three-dimensional T1-weighted sequence (repetition time [TR] = 2530 ms, echo time [TE] = 3 ms, echo spacing = 7.25 ms, flip angle [FA] = 7 degrees, field of view = 256 × 256 mm, voxel size = 1 × 1 × 1 mm). Furthermore, resting-state functional MR images were obtained through a T2*-weighted gradient-echo approach, echo-planar sequence (TR = 2500 ms, TE = 30 ms, FA = 90 degrees, and voxel size = 3.5 × 3.5 × 3.5 mm). Two hundred MRI volumes of each subject were obtained with their eyes closed. A functional whole-brain image volume comprised 43 interleaved horizontal slices, all parallel with the intercommissural plane. Furthermore, the acquired T1-weighted images provided better correction for the anatomical interpretation from functional analysis.

### Preprocessing and feature extraction for structural and resting-state functional MRI

First, the cortical and subcortical structures were determined using Freesurfer (version 6.0, https://surfer.nmr.mgh.harvard.edu) as follows: affine registration with the MNI305 space, B_1_ bias field correction, skull stripping, cortical surface reconstruction, gray and white matter segmentation, high-dimensional nonlinear alignment to the MNI305 template, and brain region labeling. Furthermore, when using Freesurfer, a more precise skull-stripping algorithm, namely HD-BET^53^, based on an artificial neural network, replaced the watershed algorithm. Subsequently, the volume of the subcortical regions and the volume and thickness of cortical regions were determined using the Desikan–Killiany atlas^54^. Second, preprocessing of the functional imaging data was performed using Statistical Parametric Mapping (SPM12, Wellcome Institute of Neurology, University College London, UK, https://www.fil.ion.ucl.ac.uk/spm/) with MATLAB 2019b (MathWorks, Natick, MA, USA) as follows: exclusion of the initial eight volumes, compensation for the slice-dependent time shifts, correction for head motion, coregistration of functional imaging volumes with their own anatomical images, spatial normalization into the Montreal Neurological Institute space using a nonlinear warping algorithm with resampling at a voxel size of 3 × 3 × 3 mm^3^, elimination of spurious data utilizing the Friston 24-parameter model^55^ and data including white matter signals, cerebrospinal fluid signals, and global signals, band-pass filtering from 0.01 to 0.08 Hz, and smoothing using a 4-mm full-width half-maximum Gaussian kernel. In addition, the participants with a mean framewise displacement > 0.2 mm were removed. Furthermore, global signals and group framewise displacement were regressed out for eliminating the impact of motion^56,57^. Thereafter, functional connectivity maps were constructed in accordance with Shen’s whole-brain functional-connectivity-based atlas^58^, parcellating the whole brain into 268 regions comprising eight networks: the MFN, FPN, DMN, SC, MON, the visual I network (VisI), the visual II network (VisII), and the visual association network (VA). The functional connectivity of each pair across the 268 regions was examined using Pearson’s correlation coefficient and converted through Fisher’s *r*-to-*z* transformation. Both the anatomical and functional images were controlled by age and sex. Furthermore, after rejecting data for participants with substantial head motion (mean framewise displacement > 0.2 mm), 98 patients with BD were recruited—specifically 51 patients with BDI, 43 patients with BDII, and 4 patients with a mixed phenotype, data for whom were used in successive analyses performed using MATLAB 2019b on a PC equipped with an i7-7700 CPU, and a RAM with 16 GB.

### Feature selection and model training for classifying fewer and more occurrences groups with each clinical pattern and diagnostic subtypes

To assess the discriminative capacity of the multiple neurobiological factors for patient clinical patterns at an individual level, the patients were binomially divided into fewer and more occurrences groups in accordance with the number of episodes and hospitalizations and their history of suicide and psychosis. Thereafter, to assess the classification performance and the neurobiological markers used to differentiate between the fewer and more occurrences groups, considering various aspects of clinical patterns and comparing between the diagnostic subtypes (i.e., BDI and BDII), we classified the two subgroups with each clinical pattern and the two diagnostic subtypes. Because the features of the BD subgroups were more similar between subgroups than with HCs but a relatively small sample size for each group was available, generalized features were estimated by bootstrapping 90% of total patients (i.e., 88 patients) 1000 times (see Supplementary Fig. S1). The bootstrapping method is a resampling method to independently obtain samples of the same size from existing data with replacement and computing statistics of these resampled data. This method utilizes estimations of the population parameters associated with the convergence of probability. Through the bootstrapping method, the more relevant and less redundant features for these 1000 estimated resampled groups were selected through mRMR. MRMR uses mutual information (*I*) to select the features displaying minimal redundancy and maximal relevance to the category being investigated. The importance of the feature subsets was determined from the mutual information quotient (*MIQ*) value of each feature as follows:

1 $${MIQ}_{x}=\frac{{V}_{x}}{{W}_{x}}$$

where ${V}_{x}$ is the mutual information of feature $x$ and the response, and ${W}_{x}$ is the sum of the mutual information of feature $x$ and other features. A high *MIQ* value represents a feature with higher relevance to the response and lower relevance to all the other features. Thereafter, from among these 1000 sets of selected features, those accumulated beyond the thresholds, including features more likely to be selected from the population through mRMR, were utilized to train support vector machine (SVM) with radial basis function. Each model with a specific accumulated feature set was trained 100 times by using the model of threefold nested cross-validation, thus displaying robust performance, with tenfold cross-validation for the training set. All participants were segregated into three folds, one used as the testing set in turn; the other two, the training set. To improve generalization, important features for each training set were selected by mRMR from the features selected by bootstrapping. After each fold was used as a testing set, the overall performance indicators of the classification, including accuracy, sensitivity, specificity, and AUC, were averaged. The selected features, accumulated beyond the specific threshold and generated the most optimal mean performance, were utilized for the following analyses. The performance of classifying pairs of subgroups was compared by using independent samples *t* tests.

### Multiple linear regression between cognition and features for classification of subtypes

To examine the proportion of variance in cognitive performance explained by the selected features of paired subgroups, the WLT I and II, the subtests of Wechsler Memory Scale-III^59^, were utilized to measure verbal memory, including immediate and delayed free recall, learning slope, recognition, and retention of lists of words. The scaled scores of each task were recorded. Then, the multiple linear regression analysis was conducted for WLT scores and the features selected after bootstrapping with the threshold of the most optimal mean performance and the proportion of variance (*R*^2^) explained was computed.

Effects of confounding factors on the classification.

The effects of clinical confounding factors, including disease duration, symptoms, mood states, and medication, were estimated form the association between these factors and the features repeatedly selected through mRMR during nested cross-validation. For continuous variables including duration and symptom scores, Pearson’s correlation coefficients were used; for categorical variables including mood states, patient groups with or without atypical antipsychotics, patient groups with or without antidepressants, and those with or without mood stabilizers were subjected to analysis of variance and independent samples *t* tests.

## Supplementary Information

Supplementary Information.

## Data Availability

All data analyzed during this study are included in this published article^60^. Processed data are avalible from the corresponding author on reasonable request.

## Code availability

In-house script is available publicly at https://github.com/Mantouling/BDclassification.git.
