# Supplementary material for: Identifying subtypes of bipolar disorder based on clinical and neurobiological characteristics
Source: Sci Rep. 2021 Aug 24;11:17082. doi: 10.1038/s41598-021-96645-5 (PMC8385023; doi:10.1038/s41598-021-96645-5)
Supplement: Supplementary file 1 — Supplementary Information. [file 41598_2021_96645_MOESM1_ESM.docx]

**Title: Identifying Subtypes of Bipolar Disorder Based on Clinical and Neurobiological Characteristics**

**Yen-Ling Chen^a,b,1^, Pei-Chi Tu^c,d,e,f^, Tzu-Hsuan Huang^a,b^, Ya-Mei Bai^d,e^, Tung-Ping Su^d,e,g^, Mu-Hong Chen^d,e^, Yu-Te Wu^a,b*^**

^a^ Institute of Biophotonics, National Yang Ming Chiao Tung University, Taipei 112, Taiwan

^b^ Brain Research Center, National Yang Ming Chiao Tung University, Taipei 112, Taiwan

^c^ Department of Medical Research and Education, Taipei Veterans General Hospital, Taipei 112, Taiwan

^d^ Department of Psychiatry, Taipei Veterans General Hospital, Taipei 112, Taiwan

^e^ Division of Psychiatry, Faculty of Medicine, National Yang Ming Chiao Tung University, Taipei 112, Taiwan

^f^ Institute of Philosophy of Mind and Cognition, National Yang Ming Chiao Tung University, Taipei 112, Taiwan

^g^ Department of Psychiatry, Cheng-Hsin General Hospital, Taipei 112, Taiwan

*** Correspondence:**

Yu-Te Wu

Institute of Biophotonics, National Yang Ming Chiao Tung University, No.155, Sec.2, Linong St., Taipei 112, Taiwan

+886-2-28267000 #66138

ytwu@ym.edu.tw

**Material and Methods**

*Schematic representation of the present study*


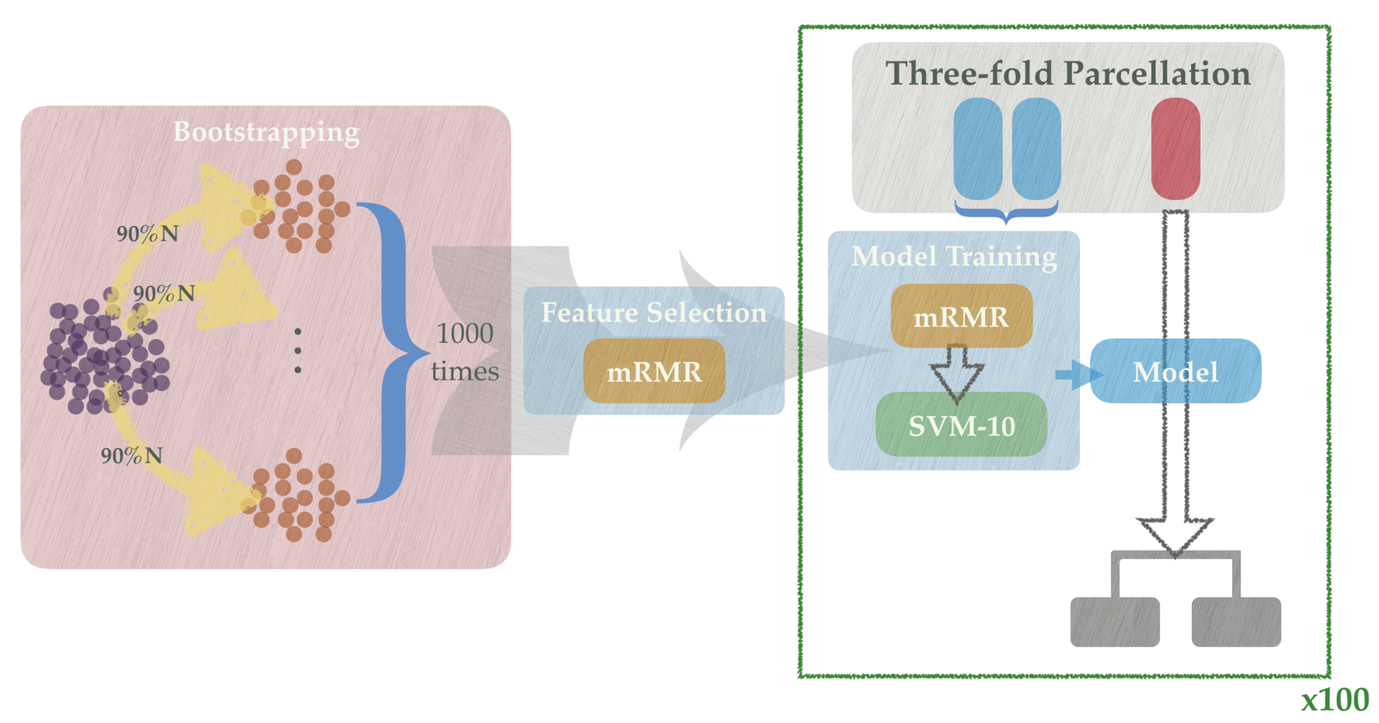
Figure S1. Schematic representation of the classification of clinical patterns; mRMR, minimum redundancy maximum relevance; SVM-10, support vector machine with 10-fold cross-validation.

**Results**

Table S1 summarizes additional results of the comparison of demographic data among pairs of fewer and more occurrences groups, along with diagnostic subtypes.

| Table S1. Demographic data of groups with fewer and more occurrences of clinical patterns and diagnostic subtypes | | | | | | | |
| --- | --- | --- | --- | --- | --- | --- | --- |
|  | Fewer occurrences group | | | More occurrences group | | | *p*-value |
| ***The number of episodes*** |  | | |  | | |  |
| Duration | 8.09 | ± | 7.847 | 12.84 | ± | 9.718 | 0.0106* |
| Education (year) | 13.77 | ± | 2.814 | 13.58 | ± | 3.331 | 0.7803 |
| Mood state |  |  |  |  |  |  | 0.4023 |
| Euthymic (%) | 48.8 | | | 32.7 | | |  |
| Hypomanic (%) | 4.7 | | | 7.3 | | |  |
| Depressed (%) | 41.8 | | | 50.9 | | |  |
| Mixed (%) | 4.7 | | | 9.1 | | |  |
| Medication |  |  |  |  |  |  |  |
| Atypical antipsychotics (%) | 75.0 | | | 73.2 | | | 0.8713 |
| Antidepressants (%) | 33.3 | | | 51.2 | | | 0.1615 |
| Mood stabilizers (%) | 70.8 | | | 68.3 | | | 0.8304 |
| ***The number of hospitalizations*** |  | | |  | | |  |
| Duration | 9.11 | ± | 9.168 | 14.23 | ± | 9.291 | 0.0241* |
| Education (year) | 13.94 | ± | 3.553 | 12.61 | ± | 3.052 | 0.1152 |
| Mood state |  |  |  |  |  |  | 0.3503 |
| Euthymic (%) | 17.6 | | | 51.4 | | |  |
| Hypomanic (%) | 5.9 | | | 11.4 | | |  |
| Depressed (%) | 64.7 | | | 31.4 | | |  |
| Mixed (%) | 11.8 | | | 5.8 | | |  |
| Medication |  |  |  |  |  |  |  |
| Atypical antipsychotics (%) | 52.9 | | | 87.1 | | | 0.0090* |
| Antidepressants (%) | 64.7 | | | 29.0 | | | 0.0165* |
| Mood stabilizers (%) | 76.5 | | | 67.7 | | | 0.5246 |
| ***Whether attempting suicide*** |  | | |  | | |  |
| Duration | 10.40 | ± | 10.080 | 11.17 | ± | 8.162 | 0.6818 |
| Education (year) | 13.90 | ± | 3.454 | 13.38 | ± | 2.650 | 0.4278 |
| Mood state |  |  |  |  |  |  | 0.0118* |
| Euthymic (%) | 47.2 | | | 31.1 | | |  |
| Hypomanic (%) | 3.8 | | | 8.9 | | |  |
| Depressed (%) | 43.4 | | | 51.1 | | |  |
| Mixed (%) | 5.6 | | | 8.9 | | |  |
| Medication |  |  |  |  |  |  |  |
| Atypical antipsychotics (%) | 71.4 | | | 76.7 | | | 0.6319 |
| Antidepressants (%) | 28.8 | | | 63.3 | | | 0.0049* |
| Mood stabilizers (%) | 71.4 | | | 66.7 | | | 0.6784 |
| ***Whether having the history of psychosis*** | | | |  | | |  |
| Duration | 9.82 | ± | 9.061 | 11.59 | ± | 9.400 | 0.3532 |
| Education (year) | 13.80 | ± | 3.368 | 13.47 | ± | 2.880 | 0.6165 |
| Mood state |  |  |  |  |  |  | 0.9491 |
| Euthymic (%) | 37.2 | | | 42.6 | | |  |
| Hypomanic (%) | 7.0 | | | 5.6 | | |  |
| Depressed (%) | 48.8 | | | 44.4 | | |  |
| Mixed (%) | 7.0 | | | 7.4 | | |  |
| Medication |  |  |  |  |  |  |  |
| Atypical antipsychotics (%) | 68.4 | | | 79.5 | | |  |
| Antidepressants (%) | 50.0 | | | 41.0 | | |  |
| Mood stabilizers (%) | 73.1 | | | 66.7 | | |  |
|  | BDI | | | BDII | | |  |
| Duration | 12.48 | ± | 9.832 | 8.80 | ± | 7.741 | 0.0502 |
| Education (year) | 13.61 | ± | 3.137 | 13.69 | ± | 3.166 | 0.9035 |
| Mood state |  |  |  |  |  |  | 0.1650 |
| Euthymic (%) | 49.0 | | | 30.2 | | |  |
| Hypomanic (%) | 7.8 | | | 4.7 | | |  |
| Depressed (%) | 35.4 | | | 58.1 | | |  |
| Mixed (%) | 7.8 | | | 7.0 | | |  |
| Medication |  |  |  |  |  |  |  |
| Atypical antipsychotics (%) | 75.7 | | | 73.1 | | | 0.8155 |
| Antidepressants (%) | 27.0 | | | 69.2 | | | 0.0009* |
| Mood stabilizers (%) | 67.6 | | | 69.2 | | | 0.8890 |

* *p* < 0.05

BDI: bipolar type 1 disorder; BDII: bipolar type 2 disorder.

Table S2 indicates the major features for classifying mild and severe groups of four different clinical patterns and for BDI and BDII. Since Shen’s parcellation is not restricted by anatomical brain structure, that is by the gyrus and sulcus, the centroid location of the parcellated region was used to provide more information about the regions.

| Table S2. The major features of the classification analyses for the clinical patterns | | | | | | | | | |
| --- | --- | --- | --- | --- | --- | --- | --- | --- | --- |
| Regions 1 (with region label) | | | |  | Regions 2 (with region label) | | | | |
| ***The number of episodes*** | | | |  |  |  |  | |  |
| Severe group > Mild group | | | |  |  |  |  | |  |
| 80 | Right calcarine | in | VisI | – | 26 | Right superior frontal cortex | | in | MON |
| 134 | Left anterior cingular cortex | in | DMN | – | 120 | Right caudate | | in | SC |
| 158 | Left postcentral cortex | in | MON | – | 10 | Right superior medial frontal cortex | | in | MFN |
| 179 | Left postcentral cortex | in | MON | – | 100 | Right crus II of cerebellum | | in | VisII |
| 223 | Left posterior cingular cortex | in | DMN | – | 10 | Right superior medial frontal cortex | | in | MFN |
| 230 | Left hippocampus | in | SC | – | 127 | Right thalamus | | in | SC |
| Mild group > Severe group | | | |  |  |  | |  |  |
| 26 | Right superior frontal cortex | in | MON | – | 19 | Right middle frontal cortex | | in | FPN |
| 84 | Right middle cingular cortex | in | MON | – | 43 | Right angular gyrus | | in | VA |
| 85 | Right posterior cingular cortex | in | DMN | – | 7 | Right middle frontal cortex | | in | FPN |
| 86 | Right precuneus | in | DMN | – | 10 | Right superior medial frontal cortex | | in | MFN |
| 101 | Right lobule IV,V of cerebellum | in | SC | – | 51 | Right middle temporal pole | | in | MON |
| 108 | Right lobule IX of cerebellum | in | SC | – | 19 | Right middle frontal cortex | | in | FPN |
| 115 | Right lobule IX of cerebellum | in | DMN | – | 19 | Right middle frontal cortex | | in | FPN |
| 167 | Left postcentral cortex | in | MON | – | 100 | Right crus II of cerebellum | | in | VisII |
| 172 | Left postcentral cortex | in | MON | – | 19 | Right middle frontal cortex | | in | FPN |
| 177 | Left superior parietal cortex | in | VA | – | 164 | Left middle frontal cortex | | in | FPN |
| 203 | Left middle occipital cortex | in | DMN | – | 172 | Left postcentral cortex | | in | MON |
| 223 | Left posterior cingular cortex | in | DMN | – | 7 | Right middle frontal cortex | | in | FPN |
| 223 | Left posterior cingular cortex | in | DMN | – | 141 | Left superior frontal cortex | | in | DMN |
| 227 | Left posterior cingular cortex | in | DMN | – | 7 | Right middle frontal cortex | | in | FPN |
| ***The number of hospitalizations*** | | | |  |  |  | |  |  |
| Severe group > Mild group | | | |  |  |  | |  |  |
| 46 | Right superior temporal cortex | in | MON | – | 23 | Right postcentral cortex | | in | MON |
| 137 | Left rectus | in | MFN | – | 119 | Right lobule IV,V of cerebellum | | in | SC |
| 140 | Left superior medial frontal cortex | in | MFN | – | 132 | Right midbrain | | in | SC |
| 160 | Left precentral cortex | in | MON | – | 43 | Right angular gyrus | | in | VA |
| 207 | Left lingual gyrus | in | VisI | – | 133 | Right pons | | in | SC |
| Mild group > Severe group | | | |  |  |  | |  |  |
| 90 | Right precuneus | in | DMN | – | 37 | Right insula | | in | MON |
| 102 | Right crus I of cerebellum | in | VisII | – | 97 | Right parahippocampus | | in | MON |
| 147 | Left inferior triangular frontal cortex | in | FPN | – | 110 | Right lobule VI of cerebellum | | in | SC |
| 187 | Left middle temporal pole | in | MFN | – | 162 | Left supplementary motor area | | in | MFN |
| 248 | Left lobule VI of cerebellum | in | SC | – | 147 | Left inferior triangular frontal cortex | | in | FPN |
| ***Whether attempting suicide*** | | | |  |  |  | |  |  |
| Severe group > Mild group | | | |  |  |  | |  |  |
| 59 | Right fusiform gyrus | in | VA | – | 45 | Right supramarginal cortex | | in | MON |
| 157 | Left inferior opercular frontal cortex | in | FPN | – | 16 | Right inferior triangular frontal cortex | | in | MFN |
| 174 | Left paracentral lobule | in | MON | – | 33 | Right precentral cortex | | in | MON |
| 185 | Left inferior temporal cortex | in | MFN | – | 25 | Right supplementary motor area | | in | MON |
| 188 | Left superior temporal pole | in | MON | – | 140 | Left superior medial frontal cortex | | in | MFN |
| 196 | Left inferior temporal cortex | in | FPN | – | 110 | Right lobule VI of cerebellum | | in | SC |
| 198 | Left fusiform gyrus | in | VisI | – | 180 | Left superior temporal cortex | | in | MON |
| 204 | Left middle occipital cortex | in | VA | – | 69 | Right inferior temporal cortex | | in | VA |
| 255 | Left lobule IV,V of cerebellum | in | SC | – | 159 | Left precentral cortex | | in | MON |
| Mild group > Severe group | | | |  |  |  | |  |  |
| 107 | Right crus II of cerebellum | in | SC | – | 39 | Right postcentral cortex | | in | MON |
| 121 | Right caudate | in | SC | – | 94 | Right hippocampus | | in | SC |
| 151 | Left inferior orbitofrontal cortex | in | MFN | – | 73 | Right superior occipital cortex | | in | VA |
| 160 | Left precentral cortex | in | MON | – | 139 | Left superior orbitofrontal cortex | | in | FPN |
| 170 | Left insula | in | MON | – | 63 | Right middle temporal cortex | | in | MON |
| 199 | Left inferior temporal cortex | in | FPN | – | 72 | Right fusiform gyrus | | in | VisI |
| 253 | Left crus I of cerebellum | in | SC | – | 214 | Left inferior occipital cortex | | in | VisII |
| - The volume of the right frontal pole (the feature extracted from structural images). | | | | | | | | | |
| ***Whether having psychosis*** | | | |  |  |  | |  |  |
| Severe group > Mild group | | | |  |  |  | |  |  |
| 53 | Right middle temporal pole | in | MFN | – | 8 | Right inferior triangular frontal cortex | | in | FPN |
| 61 | Right superior temporal cortex | in | MON | – | 57 | Right inferior temporal cortex | | in | MFN |
| 109 | Right lobule VIII of cerebellum | in | MON | – | 44 | Right precuneus | | in | SC |
| 109 | Right lobule VIII of cerebellum | in | MON | – | 108 | Right lobule IX of cerebellum | | in | SC |
| 131 | Right pons | in | SC | – | 42 | Right precuneus | | in | VisI |
| 207 | Left lingual gyrus | in | VisI | – | 160 | Left precentral cortex | | in | MON |
| 214 | Left inferior occipital cortex | in | VisII | – | 130 | Right pons | | in | SC |
| 243 | Left lobule IX of cerebellum | in | SC | – | 109 | Right lobule VIII of cerebellum | | in | MON |
| 247 | Left crus III of cerebellum | in | FPN | – | 218 | Left middle cingular cortex | | in | MON |
| 259 | Left caudate | in | SC | – | 204 | Left middle occipital cortex | | in | VA |
| Mild group > Severe group | | | |  |  |  | |  |  |
| 32 | Right middle frontal cortex | in | SC | – | 12 | Right superior frontal cortex | | in | MFN |
| 56 | Right inferior temporal cortex | in | MFN | – | 8 | Right inferior triangular frontal cortex | | in | FPN |
| 139 | Left superior orbitofrontal cortex | in | FPN | – | 83 | Right anterior cingular cortex | | in | SC |
| 180 | Left suporior temporal cortex | in | MON | – | 79 | Right lingual gyrus | | in | VisI |
| 196 | Left inferior temporal cortex | in | FPN | – | 136 | Left rectus | | in | SC |
| 204 | Left middle occipital cortex | in | VA | – | 49 | Right angular gyrus | | in | DMN |
| 204 | Left middle occipital cortex | in | VA | – | 90 | Right precuneus | | in | DMN |
| 229 | Left hippocampus | in | SC | – | 35 | Right insula | | in | MON |
| 250 | Left lobule VIII of cerebellum | in | SC | – | 151 | Left inferior orbitofrontal cortex | | in | MFN |
| ***Diagnostic subtypes*** | | | |  |  |  | |  |  |
| BDI > BDII | | | |  |  |  | |  |  |
| 85 | Right posterior cingular cortex | in | DMN | – | 23 | Right postcentral cortex | | in | MON |
| 101 | Right lobule IV,V of cerebellum | in | SC | – | 29 | Right supplementary motor area | | in | SC |
| 135 | Left inferior orbitofrontal cortex | in | SC | – | 6 | Right superior frontal cortex | | in | DMN |
| 167 | Left postcentral cortex | in | MON | – | 145 | Left superior medial frontal cortex | | in | MFN |
| 197 | Left middle temporal cortex | in | MON | – | 103 | Right lobule III of cerebellum | | in | SC |
| 227 | Left posterior cingular cortex | in | DMN | – | 23 | Right postcentral cortex | | in | MON |
| 228 | Left amygdala | in | MON | – | 6 | Right superior frontal cortex | | in | DMN |
| 228 | Left amygdala | in | MON | – | 79 | Right lingual gyrus | | in | VisI |
| 246 | Left crus II of cerebellum | in | FPN | – | 9 | Right middle frontal cortex | | in | FPN |
| 254 | Left lobule VI of cerebellum | in | SC | – | 28 | Right superior medial frontal cortex | | in | SC |
| 265 | Left midbrain | in | SC | – | 16 | Right inferior triangular frontal cortex | | in | MFN |
| BDII > BDI | | | |  |  |  | |  |  |
| 25 | Right supplementary motor area | in | MON | – | 20 | Right insula | | in | SC |
| 88 | Right middle cingular cortex | in | SC | – | 49 | Right angular gyrus | | in | DMN |
| 158 | Left postcentral cortex | in | MON | – | 145 | Left superior medial frontal cortex | | in | MFN |
| 173 | Left insula | in | MON | – | 100 | Right crus II of cerebellum | | in | VisII |
| 198 | Left fusiform gyrus | in | VisI | – | 45 | Right supramarginal cortex | | in | MON |
| 220 | Left middle cingular cortex | in | SC | – | 197 | Left middle temporal pole | | in | MON |
| 228 | Left amygdala | in | MON | – | 94 | Right hippocampus | | in | SC |
| 233 | Left parahippocampus | in | SC | – | 230 | Left hippocampus | | in | SC |
| 242 | Left crus II of cerebellum | in | FPN | – | 173 | Left insula | | in | MON |

DMN: default mode network; FPN: frontoparietal network; MFN: medial frontal network; MON: motor network; SC: subcortical and cerebellar network; VA: visual association network; VisI: visual I network; VisII: visual II network
